# Editorial for Special Issue “Natural Products as Potential Source of Antidiabetic Compounds”

**DOI:** 10.3390/cimb45040176

**Published:** 2023-03-24

**Authors:** Hidayat Hussain

**Affiliations:** Department of Bioorganic Chemistry, Leibniz Institute of Plant Biochemistry, Weinberg 3, 06120 Halle (Saale), Germany; hidayat.hussain@ipb-halle.de

Natural products (NPs) are characterized by possessing intriguing scaffold diversity along with structural complexity and have been a comprehensive source of lead compounds for drug discovery. Furthermore, plants, bacteria, and fungi provide NPs with exciting chemical diversity, possessing significant biological and pharmacological effects. Notably, NP-based lead molecules are safe and cheaper. Diabetes mellitus is considered to be one of the more severe healthcare issues of modern times. Moreover, its prevalence is clearly demonstrated by the increasing number of diabetic patients from 537 million in 2021 to a projected 783 million in 2045 [1]. Many scientists around the globe have published in this Special Issue (SI), which includes 10 papers that provide the readers of *Current Issues in Molecular Biology* an updated and new perspective on natural products in antidiabetic drug discovery.

Alfadda and co-workers [1] studied the urinary proteome differences in type 2 diabetes patients. The protein profile in patients was studied using mass spectrometry. The investigation demonstrated that after a twelve-week treatment with liraglutide, the patients had improved glycemic control and altered urinary protein abundance. Serotransferrin, albumin, keratins K1 and K10, and MT-2 were upregulated, and the abundance of ZAG and α1-AT decreased. Additionally, a significant change in HbA1c was found with no changes in body weight or markers of dyslipidemia. The authors suggested that the enhancement of MT-2 and reduction in α1-AT and ZAG could be due to the renoprotective effect of liraglutide.

The fruits, leaves, and roots of *Solanum indicum* were traditionally employed to treat asthma, rhinitis, cough, hiccups, sore throat, abdominal pain, sexual disorders, worm infestation, inflammation, fever, urinary complications, insomnia, cardiac weakness, and blood disorders. It has been reported that the crude extracts of various *Solanum* species exhibit in vitro antidiabetic effects [2,3,4]. Gadewar et al. [5] demonstrated that the oral administration of *S. indicum* fruit extracts significantly reduced blood glucose levels at a dose of 200 mg/kg. In addition, the fruit extract intake resulted in α-glycosidase and α-amylase inhibition. Furthermore, it significantly decreased triglyceride and serum cholesterol levels in diabetic rats. Significantly, this extract prevented the oxidative stress associated with type II diabetes.

*Lippia alba* has been traditionally reported to exhibit antifungal, antidiarrheal, analgesic, and anticholesterolemic properties [6]. In addition, *Lippia* species have been reported to affect adipocyte functionality via reversing the hypertrophic state, thereby reducing lipogenesis and altering adipogenesis and lipolysis [6]. Moreno-Castellanos and co-workers [7] evaluated the activity of *Lippia alba* essential oil (EO) on lipid mobilization, viability, and the adipogenesis of adipocytes. The results illustrated that *L. alba* carvone chemotype EO did not reduce adipocyte viability. Moreover, *L. alba* EO caused changes in adipogenesis and lipid mobilization, which resulted in the reversal of adipocyte hypertrophy. The authors postulated that this effect of *L. alba* EO could be due to effects on lipolytic and lipogenic pathways along with modifications in the expression of adipogenesis genes.

Insulin resistance is a major issue in diabetes and is mostly connected to diabetes mellitus type 2 (T2DM) and obesity. However, young people diagnosed with type 1 diabetes mellitus (T1DM) are either obese or overweight at the time of diagnosis [8]. Flotynska et al. [8] evaluated NADH dehydrogenase [ubiquinone] iron–sulfur protein 8 (NDUFS8) serum concentrations along with their relationship with insulin resistance in T1DM. The study group comprised 24 men and 12 women. The results revealed that the group with higher insulin sensitivity also had a high serum concentration of NDUFS8 protein. The study concluded that high concentrations of NDUFS8 protein serum were linked with higher insulin sensitivity among T1DM patients.

El-Megharbel and co-workers [9] prepared Co(II) and Mn(II) complexes of the antidiabetic drug sitagliptin (STG). The complexes were characterized using FT-IR, DG/TG, XRD, ESM, and TEM. The spectroscopic data demonstrated that STG acts as a bidentate ligand, and the Mn/STG metal complex exhibits a square planner geometry. The biological results confirmed that the Co/STG and Mn/STG complexes were efficient in the treatment of oxidative stress and hepatic alterations and additionally improved the histological structure of liver tissues. Furthermore, the STG/Mn complex demonstrated significant antimicrobial effects towards *Streptococcus pneumonia* and *Bacillus subtilis*, while the STG/Co complex was strongly effective towards *Pseudomonas aeruginosa*, *Staphylococcus aureus*, and *Escherichia coli*.

Drug–drug interactions (DDIs) produced via the co-administration of multiple drugs are crucial safety issues to be considered in treatment regimens, and any possible consequences, such as loss of efficacy or toxicity, have to be clearly identified. Thus, safe and effective drug therapy remains a challenging issue in the clinic. Various drugs have been withdrawn from the drug market due to serious DDIs, which include mibefradil, terfenadine, and cerivastatin. Hamza et al. [10] evaluated the DDIs between etoricoxib and azithromycin and administered ascorbic acid intraperitoneally to male rats in order to identify any adverse reactions resulting from the treatment. The authors demonstrated that ascorbic acid had significant hepatic, antioxidant, and cardiac ameliorative effects and can alleviate drug interaction toxicity.

The powder from the root of *Calotropis procera* is employed to treat asthma, eczema, bronchitis, leprosy, and elephantiasis, while the plant latex is used for the treatment of baldness, vertigo, hair loss, intermittent fevers, rheumatoid/joint swellings, toothache, and paralysis. Furthermore, leaf extracts are used to reduce swelling and joint pain, and various plant extracts of *C. procera* possess antidiabetic properties. Kwofie and co-workers [11] employed molecular docking protocols in order to screen compounds isolated from *C. procera* for binding to receptor α-glucosidases. Syriogenin, taraxasterol, calotoxin, and isorhamnetin-3-*O*-robinobioside were identified as lead molecules with binding energies of −35.1, −40.2, −34.3, and −34.3 kJ/mol towards α-glucosidase, respectively.

Krawczyk et al. [12] published a meta-analysis based on evidence from critical studies in which they pointed out the antidiabetic effect of polyphenol-rich extracts from *Momordica charantia*, *Gymnema montanum*, and *Moringa oleiferadata*. The authors analyzed the effectiveness of oral supplementation of the three selected plant extracts in animal models and evaluated their effects on both oxidative stress and physiological parameters. For this review, 23 published articles were selected that studied the in vitro and in vivo antidiabetic potential of these extracts. This meta-analysis demonstrated the following parameter changes, which resulted from an investigation of the supplementary information: (i) decreased insulin resistance, (ii) reduced oxidative stress, (iii) reduced adiposity, (iv) modulatory effects on glycolysis, (v) increased insulin release, (vi) decreased fasting blood glucose, (vii) diabetes-associated weight loss, and (viii) lowered oxidative status.

Pancreatic β-cell destruction can be caused either by the absence of insulin or an insulin shortage, resulting in the development of diabetes mellitus in patients. Many trials have been conducted since the 19th century for the effective treatment of diabetes. In early times, diabetes was treated by taking insulin derived from an animal’s extract. However, this therapy caused some consequential allergic reactions. Thus, scientists around the globe have concentrated their efforts on establishing alternative and more effective strategies to treat diabetes. Hanif et al. [13] published a review about the proteomic changes and discovery of engineered insulin. In their review, they discussed how various scientists had formulated insulin derivatives that mimic the natural human insulin via recombinant DNA technology. The authors addressed various critical questions, including how insulin is prepared and what the positive and negative impacts insulin derivatives have on T1DM and T2DM patients. Moreover, the authors discussed various new strategies that have been established for the delivery of insulin. Currently, various investigations remain a priority and are underway to further upgrade insulin-based treatments.

McCarty et al. [14] published a review addressing nutraceuticals that can be used to prevent diabetic complications, with a focus on dicarbonyl and oxidative stress. They discussed various nutraceuticals used to control oxidative and dicarbonyl stress in order to prevent diabetes-related complications. For instance, berberine, which is a nutraceutical isolated from *Coptis chinensis*, is employed in traditional Chinese medicine to control diabetes. Other nutraceuticals that can be used to reduce oxidative and dicarbonyl stress include resveratrol, ferulic acid, tetrahydrocurcumin, urolithin A, curcumin, hymoquinone, spermidine, astaxanthin, and capsaicin.

## Data Availability

Not applicable.
